# Effect of four trace elements on *Paenibacillus polymyxa* Pp-7250 proliferation, activity and colonization in *ginseng*

**DOI:** 10.1186/s13568-018-0694-0

**Published:** 2018-10-11

**Authors:** Yugang Gao, Jing Liang, Ruxue Xiao, Pu Zang, Yan Zhao, Lianxue Zhang

**Affiliations:** College of Traditional Chinese Medicine Materials, Jilin Agricultural University, Changchun, 130118 China

**Keywords:** Trace elements, *Paenibacillus polymyxa* Pp-7250, Proliferation, Ginsenoside, Soil microbial colonization

## Abstract

Trace elements are essential nutrients for the growth of microorganisms and play an important role in their proliferation. Hence, the purpose of this paper is to explore the optimal C and N sources for large-scale culture of *Paenibacillus polymyxa*, and to screen trace elements that can promote their proliferation and improve the activity. First, the concentration of *Paenibacillus polymyxa* Pp-7250, the number of spores were used as evaluation index. It was found that the four trace elements Cu^2+^, Fe^2+^, Mn^2+^, and Zn^2+^ could promote the proliferation of *Paenibacillus polymyxa* at their optimal concentrations. Next, when using wheat starch as carbon source and soybean meal as nitrogen source, it was most suitable for large-scale culture. Finally, field experiments were carried out, and it was discovered that the combination of four trace elements plus the wheat soybean meal group could significantly improve the disease prevention, growth promotion ability of Pp-7250 and its colonization in ginseng. Moreover, the ability of Pp-7250 to transform ginseng roots and leaf saponins were also significantly improved. The group also affected the rhizosphere bacterial community of ginseng and the number showed a significant promotion or inhibition.

## Introduction

*Paenibacillus polymyxa* is one of the most development potential strains of the genus *Bacillus*. And reports on its application in various aspects are increasing at home and abroad, mainly focusing on biological nitrogen fixation, promoting plant growth, developing new antibiotics and preventing plant diseases. It is a worthwhile biomaterial to develop (Ji et al. 2015; Gao et al. 2018; Finch et al. 2018; Yang et al. 2018; Midhuna et al. 2017). The industrial development and production of *Paenibacillus polymyxa* has become a hotspot due to its great potential. However, some problems exist, such as the quality of bacteria is difficult to be guaranteed when the bacteria are stored and activated; most of the medium composition of beef extract, yeast extract, peptone and other expensive raw materials, or the use of pre-processing more complex PDB medium etc. (Ding et al. 2012), which restricts the large-scale industrial production. The traditional large-scale production process of microorganisms is as follows: preservation of bacteria species, activation culture of slant bacteria species, expansion culture of shake flask, liquid fermentation of fermentation tank, pilot scale amplification culture. How to obtain the conditions of fermentation with high activity and high quantity of living bacteria is the key to production. A large number of researchers have conducted studies on the application for large-scale culture of *Bacillus*, it provides a useful reference for the large-scale fermentation and cultivation of *Paenibacillus polymyxa* (Ghasemi and Ahmadzadeh 2013; Prabakaran and Hoti 2008; Prabakaran and Balaraman 2006). Thus, how to improve the number of effective living bacteria and the quality of the bacteria and promote its proliferation by optimizing the C source and N source culture methods of *Paenibacillus polymyxa*, it is the most important thing to discuss the large-scale cultivation of *Paenibacillus polymyxa* in the future. It has practical guidance value and theoretical significance.

Studies have shown that trace elements are indispensable energy substances for the growth and reproduction of microorganisms, it’s part of a multiple enzyme activity center, by regulating the active substances in microorganisms and the activity can be enhanced (Memoli et al. 2018). With the promotion of fungicides and bacterial fertilizers in recent years, the demand for probiotics is also increasing. Trace elements are essential for the growth of microorganisms and an important component of bacteria (Blamey et al. 1999), different types and amounts of trace elements can also cause differences in microbial growth and development. Studies have found that different types of trace elements, different concentrations of the same trace elements, and combinations of different trace elements can affect the proliferation of *Bacillus* (He et al. 2014; Mahmood 2010; Ruming et al. 2003; Raza et al. 2010; Nagano et al. 2013).

In recent years, the beneficial bacteria in biological control show certain advantages in controlling plant diseases (Lazarovits et al. 2014; Sharifazizi et al. 2017; Schnider et al. 1995; Sharma et al. 2018; Park et al. 2018; Siahmoshteh et al. 2018). *Bacillus* mainly by competing with surrounding pathogenic nutrients, successfully colonizing in plants, rhizosphere, or self-secreting antibacterial active substances to prevent diseases (Chinheya et al. 2017; Mercado-Flores et al. 2014; Cheng et al. 2017; Gowtham et al. 2018; Touré et al. 2004). The addition of trace elements can significantly affect the ability of microorganisms to prevent disease and promote growth (Zhang et al. 2013; Tabbene et al. 2009; Akladious and Mohamed 2018). Therefore, the effect of the mass concentration of four trace elements on the prevention, promotion and colonization of *Paenibacillus polymyxa* Pp-7250 were determined by field experiments. Moreover, the main bacterial species of ginseng rhizosphere soil were identified by 16s rDNA gene sequence and BLAST comparison. It was of great significance to the improvement of ginseng soil, the excavation of beneficial microorganisms, and the development and utilization of ginseng-specific biocontrol agents.

## Materials and methods

### Cultivation of *Paenibacillus polymyxa* Pp-7250

*Paenibacillus polymyxa* Pp-7250 (Pp-7250) was an endophyte of ginseng and isolated from our laboratory. Preserved in the General Microbiological Center of the China Microbial Culture Collection Management Committee, Preservation number CGMCC: No. 7250. The Pp-7250 was underlined on the PDA medium with the inoculation ring, after 24 h, the single colony of Pp-7250 was moved to a conical flask with 100 mL PDB medium. To cultivate 24 h in reciprocating shaker with 28 °C, 140 r/min. The Pp-7250 culture was sterilized and saved for backup.

### Effects of single trace elements and trace elements compound on Pp-7250

The influences of trace elements on microorganism are various, and the effects of single trace elements and trace elements compound on microorganism are different. The aim of this study is to find the concentrations of trace elements, trace elements and their combinations suitable for the growth of *Paenibacillus polymyxa* (Li and Ma 2014; Trchounian et al. 2016; Ropek and Para 2002; Gupta et al. 2010). Under aseptic conditions, the PDB medium containing 5 mL different mass concentration of Cu^2+^, Fe^2+^, Mn^2+^, and Zn^2+^ was added to each sterilized test tube (Table 1). Each concentration treatment was set at 3 repetitions. Each test tube was inoculated with 0.05 mL of activated *Paenibacillus polymyxa* with an OD_600_ value of 0.5, incubated at a constant temperature of 120 r/min at 28 °C. The OD_600_ values were determined by sampling at 0, 12, 24, 36, 48, 60, 72, 84, 96, 108 and 120 h.

**Table 1:** Screening of the concentrations of four single trace elements and orthogonal table of trace elements concentration compounds

Trace elements (mg/L)	1	2	3	4	5	6	7	8
Concentration of Cu^2+^, Fe^2+^, Mn^2+^ and Zn^2+^								
Cu^2+^	0	10	30	50	100	500	2500	5000
Fe^2+^	0	10	30	50	100	500	2500	5000
Mn^2+^	0	10	30	50	100	500	2500	5000
Zn^2+^	0	50	100	500	1000	2000	3000	4000

Test number	Cu^2+^ (A)	Fe^2+^ (B)	Zn^2+^ (C)	Mn^2+^ (D)
Factors and levels of orthogonal test				
1	10	30	50	30
2	10	50	100	50
3	10	100	150	100
4	30	30	100	100
5	30	50	150	30
6	30	100	50	50
7	50	30	150	50
8	50	50	50	100
9	50	100	100	30

This article used 4 factors and 3 levels of L9 (34) orthogonal table, Cu^2+^, Fe^2+^, Mn^2+^, Zn^2+^ mass concentration as the 4 factors, see Table 1.

### Effects of different C source N source types and concentration on the number of viable bacteria and spore number of Pp-7250

Suitable culture conditions affect the activity of microorganisms. The aim of this experiment is to find the optimum medium for the growth of *Paenibacillus polymyxa* (Mosquera et al. 2014; Jeong et al. 2010; Prakasham et al. 2007; Wang et al. 2008; Jia et al. 2017; Odeniyi and Adeola 2017). Based on the PDB culture medium, different C sources (wheat starch, potato starch, sweet potato starch, corn starch, flour) were selected, respectively made into culture medium containing 5 g/L C source. The bacterial suspension was inoculated into the medium with 5% inoculation, the living bacteria and their spores were sampled and measured at 28 °C and 140 r/min cultivation conditions for 48 h. Repeated 3×. Based on the determination of the best C source species, the effects of different C source addition amounts (3 g/L, 4 g/L, 5 g/L, 6 g/L, 7 g/L) on the living bacteria and spores of *Paenibacillus polymyxa* were investigated. Different N sources (soybean, peptone, soybean meal, yeast powder, and urea) were selected to make a single nitrogen source medium. Based on the determination of the best N source species, the effects of different N source addition amounts (2 g/L, 4 g/L, 6 g/L, 8 g/L, 10 g/L) on the living bacteria and spores of *Paenibacillus polymyxa* were investigated. According to the number of viable bacteria and the number of spores, the optimum carbon nitrogen source culture medium and the optimum amount of addition were determined. In order to ensure that it can be used well in actual production, it is necessary to determine the effect of preservation time on the number of living bacteria in pp-7250. A total of 5 treatments were set up (Cu^2+^ + wheat soyabean meal, Fe^2+^ + wheat soyabean meal, Zn^2+^ + wheat soyabean meal, Mn^2+^ + wheat soyabean meal, four trace elements compound + wheat soyabean meal) in this trial. The control group were wheat starch group, soybean meal group, wheat + soybean meal group, flour group and PDB group. Stored at room temperature and pressure, the number of Pp-7250 living bacteria in the above conditions was detected every 7 days for a total of 3 months.

### Effects of four trace elements on the biocontrol of Pp-7250

First, the bacteriostatic effect of *Paenibacillus polymyxa* was explored. A total of ten treatments were set up. The control group (CK, without bacteria solution) was wheat starch group, soybean meal group, wheat plus soybean meal group, flour group, PDB group; the treatment group consisted of four trace elements compound + wheat soybean meal group, Cu^2+^ + wheat soybean meal group, Fe^2+^ + wheat soybean meal group, Mn^2+^ + wheat soybean meal group, and Zn^2+^ + wheat soybean meal group. Pipetted 20 μL of the pathogenic bacteria cultured for 12 h with a pipette and coated with a flat plate, fully coated with a sterile coating stick. Three sterilized filter paper (d = 8 mm) were placed in each petri dishes. Each filter paper was fully immersed in different treatment groups of *Paenibacillus polymyxa* for 10 min, and then put into the bacteria-coated plate. The plates coated with the bacteria solution were inverted in a constant temperature incubator and incubated at 28 °C for 24 h. The diameter of each pathogenic bacterial colony was measured by cross-crossing method, and the antibacterial rate was calculated by averaging. Bacteriostatic rate = (control group diameter of inhibition zone-treatment group inhibition zone diameter)/control group diameter of inhibition zone × 100%. At the same time, we conducted field trials. A total of 6 treatments were set for the test: The control group (CK, no bacteria solution) was PDB control group, four trace elements +PDB control group and clear water control group. The treatment group was composed of four trace elements compound +PDB group, four trace elements compound + wheat soybean meal group and PDB group. The bacterial suspension of OD_600 _= 0.5 was inoculated into the medium with 5% inoculation. This experiment was conducted in Jingyu County, Jilin Province to carry out the field validation test on the activity of *Paenibacillus polymyxa*. Before this trial, ginseng had been continuously planted in the experimental field for many years. The spraying method was stem and leaf spray, and protective row was set between treatments. The area of the control area and the treatment area were 3.33 m^2^, and the randomized block grouping was used for the test. The concentration of bacterium of 10^7^ CFU/mL was used for all plot treatments, 1×/month and 5× in total. Leaf and the growth of plants in each treatment group were observed. Five points were randomly selected in each treatment plot, a total of 10 ginseng strains were randomly selected. The plant height, stem diameter, leaf width, leaf length, petiole length, stem and leaf total dry weight and fresh weight were measured. The specific root weight of ginseng root samples were analyzed. The vertical height was measured from the base of plant stem to the highest point of new leaf and the average value was the plant height. The transverse widest part of the first blade was measured by a ruler, that was, the blade width. Stem diameter was measured with a vernier caliper, with a position of 2 cm above the first leaf blade. The whole root of ginseng was weighed by electronic balance. Then put it on the coordinate paper to measure the total length and calculate its specific root weight (root weight/root length).

### Determination of ginsenoside content

We accurately weighed 0.5 g of ginseng root and leaf samples from different treatment groups in a 10 mL centrifuge tube (PDB control group (no bacteria solution); four trace elements + PDB control group; clear water control group; four trace elements compound + PDB group; four trace elements compound + wheat soybean meal group and PDB group), added 5.0 mL chromatography methanol solution, sealed, extracted ultrasonically for 45 min, let stand overnight, then sonicated for 45 min, centrifuged, took the supernatant, filtered with 0.45 μm filter, spared. The chromatographic conditions referred to the method for simultaneous determination of 20 ginsenosides established in our laboratory (Yang et al. 2016).

### Effects of four trace elements on Pp-7250 colonization and rhizosphere bacterial community

Soil microflora is mainly represented by the number of microorganisms (bacteria, fungi and actinomycetes) in the soil. The composition of soil microorganism is closely related to the obstacle of continuous cropping, drought resistance, biological stress and salt stress (Bai et al. 2015; Cui et al. 2018). The successful application of *Bacillus* depends on its ecological interaction between soil and rhizosphere microorganisms (compatibility, symbiosis, antibiotic, competition, etc.) (Ge et al. 2015; Piromyou et al. 2013). Therefore, the interactions between *Bacillus* and rhizosphere bacteria, fungi and actinomycetes are discussed, and the changing rules of their microbial community composition in the rhizosphere are cleared, which has important guiding significance for the construction of plant healthy rhizosphere microflora. First we collected the soil samples. Rhizosphere soil samples were collected from control group and treatment group respectively, ten plants were randomly selected from each plot, and each plant was surrounded by ginseng roots by a five point sampling method. Within the range of 25 cm around the root, 0–20 cm deep soil was collected, and five points were taken and combined into one part. Each soil was sieved to remove impurities, and mixed in equal amounts. Stored at 4 °C for microbial counting. Secondly, the colonization of Pp-7250 in ginseng roots, stems leaves and soil was determined. After the root, stem and leaf of the ginseng were collected and cleaned with tap water, 0.5 g was taken and fully grinding in 5 mL 0.85% NaCl. The supernatant 1 mL was taken and diluted by 10-fold dilution method. The 10^−7^ concentration of bacterial suspension 100 μL was used to coat the solid plate, and incubated in a constant temperature incubator at 28 °C for 1 to 3 days. The number of colonies on the plate was observed and counted. Repeated 3×. The weight of soil wet was 0.5 g, and 5 mL of sterile water was added, shaking at 28 °C for 30 min, After dilution by 10-fold dilution method, the 10^−7^ concentration of bacterial suspension 100 μL was used to coat the solid plate, and incubated in a constant temperature incubator at 28 °C for 1–3 days. The number of colonies on the plate was observed and counted. Repeated 3×. Soil samples were taken to determine the colonization of Pp-7250 in soil. And the number of bacteria in each soil sample was determined by solid plate method. The weight of soil wet was 0.5 g, and 5 mL of sterile water was added, the 10^−7^ concentration of bacterial suspension 100 μL was used to coat the solid plate, and each bacterium was compared with the same amount of sterilized distilled water. The number and morphology of colonies were observed and calculated. The strains with different colony morphology were purified by plate streaking, placed in an incubator at 28 °C. After the inverted culture 1–3 days observation, the colony morphology was recorded and observed under the microscope. After purification, they were transferred to the corresponding slope for preservation and according to their morphological characteristics for the naming and classification. The soil bacteria and fungal DNA were extracted using the bacterial and fungal DNA extraction kits produced by Solarbio company, and the extracted DNA was purified and stored at − 20 °C. PCR amplification of 16s rRNA from soil bacteria and fungi was carried out by using 1492R and 27F common bacterial primers synthesized by dalian baobao biotechnology co., LTD. The PCR products were detected by 1% agarose gel electrophoresis and observed the presence of specific target bands. Then the PCR product was sent to Shanghai Shenggong Biological Engineering Technology Service Co., Ltd. for sequencing. In NCBI database, the sequence was analyzed by BLAST comparison, and the strain with the highest similarity was identified, and the biological classification status of each strain was determined.

### Statistical analysis

Sorting out data with Excel; SPSS19.0 was used for statistical analysis of the collected data.

## Results

### Effect of four trace elements on multiplication of Pp-7250

Trace elements have a certain promoting effect on the growth of *Paenibacillus polymyxa*, but it has a big relationship with its concentration. With the increase of the concentration of trace elements, its effect begins to increase. But its effect is weakened after reaching certain concentration.

**Fig. 1:**
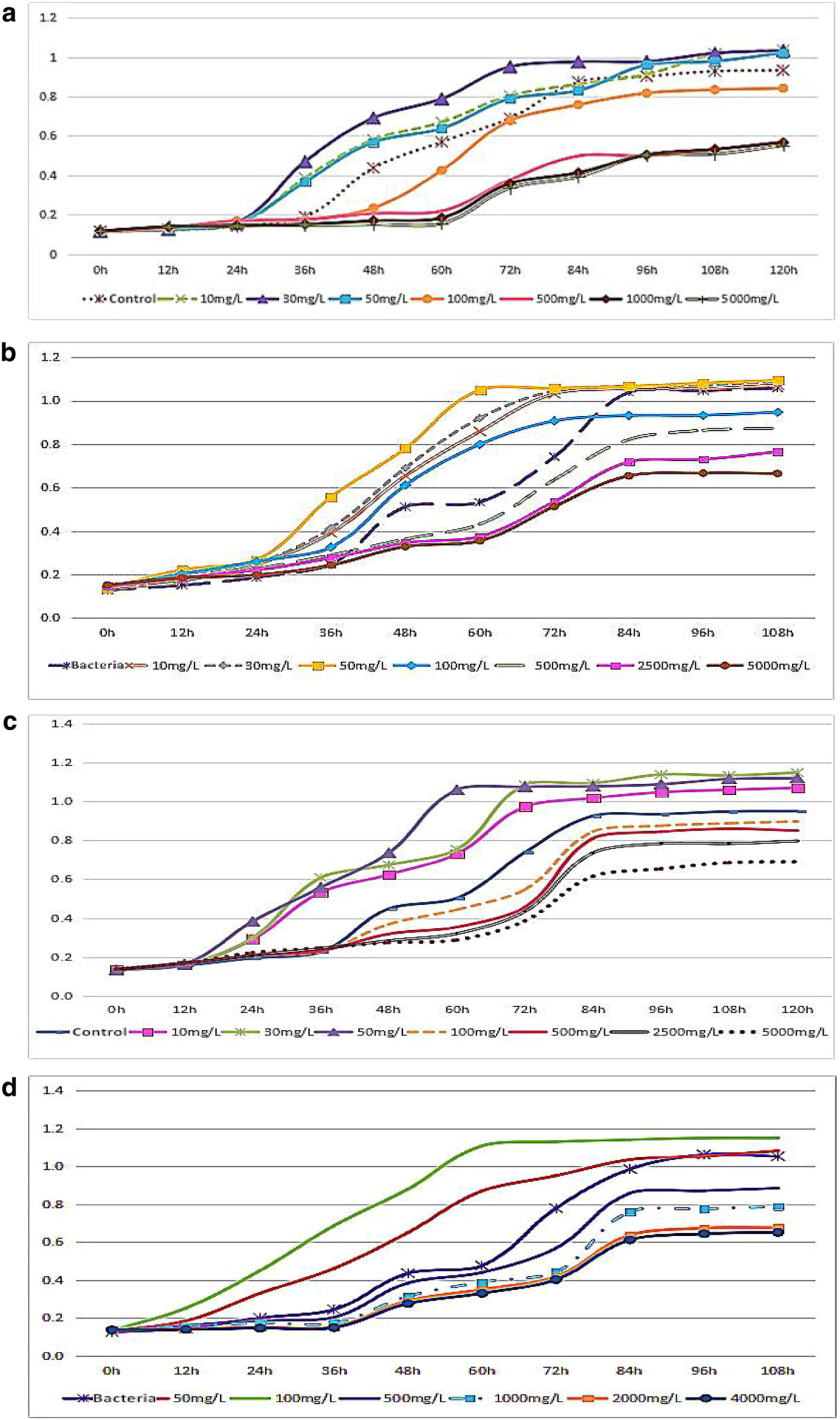
**a**–**d** The effects of Cu^2+^, Fe^2+^, Mn^2+^ and Zn^2+^ on the proliferation of Pp-7250. Take Zn^2+^ as an example. When the concentration of Zn^2+^ was 50–100 mg/L, compared with the control group, the delayed period of Pp-7250 multiplication was shortened, and the logarithmic phase was advanced; When the concentration of Zn^2+^ was 100 mg/L, the promotion of Pp-7250 was the strongest, the delayed period was shortened by 12 h, and the logarithmic stage was about 24 h in advance; The appropriate concentration of Zn^2+^ had a certain promoting effect on Pp-7250. However, when the Zn^2+^ concentration was greater than 500 mg/L, there was a certain inhibitory effect on the proliferation of Pp-7250, as the concentration increasing, the inhibitory effect increased

This study found that the concentration of Cu^2+^ at 10–50 mg/L all had the effect of promoting Pp-7250 multiplication (*P* < 0.05). Its optimal mass concentration was 30 mg/L. However, when the concentration of Cu^2+^ exceeded 100 mg/L, its multiplication was inhibited; The concentration of Fe^2+^ at 10–100 mg/L all had the effect of promoting Pp-7250 multiplication (*P *< 0.05). Its optimal mass concentration was 50 mg/L. However, when the concentration of Fe^2+^ exceeded 500 mg/L, its proliferation was inhibited; The concentration of Mn^2+^ at 10–50 mg/L all had the effect of promoting Pp-7250 multiplication (*P *< 0.05). Its optimal mass concentration was 50 mg/L. However, when the concentration of Mn^2+^ exceeded 500 mg/L, its multiplication was inhibited; The concentration of Zn^2+^ at 50–100 mg/L all had the effect of promoting Pp-7250 multiplication (*P *< 0.05). Its optimal mass concentration was 100 mg/L. However, when the concentration of Zn^2+^ exceeded 500 mg/L, its multiplication was inhibited (Fig. 1).

The single-factor experiment results found that the single trace element significantly promoted or inhibited the multiplication of Pp-7250. The concentration range of trace elements suitable for Pp-7250 multiplication was selected for orthogonal experiment. The experimental results and the variance analysis were shown in Table 2.

**Table 2:** Optimal concentration combinations of four trace elements affecting the proliferation of *Paenibacillus polymyxa* Pp-7250

Test number	Cu^2+^ (A)	Fe^2+^ (B)	Zn^2+^ (C)	Mn^2+^ (D)	OD_600_ 60 h	
Results of the orthogonal experiment						
1	1	1	1	1	0.9379	
2	1	2	2	2	1.0552	
3	1	3	3	3	0.7374	
4	2	1	2	3	0.6545	
5	2	2	3	1	0.6614	
6	2	3	1	2	1.2293	
7	3	1	3	2	0.7192	
8	3	2	1	3	0.8526	
9	3	3	2	1	0.9106	
T1	2.7305	2.3116	3.0198	2.5099	7.7581	
T2	2.5452	2.5692	2.6203	3.0037		
T3	2.4824	2.8773	2.1180	2.2445		
The average k1	0.9102	0.7705	1.0066	0.8366		
k2	0.8484	0.8564	0.8734	1.0012		
k3	0.8275	0.9591	0.7060	0.7482		
R value	0.0827	0.1886	0.3006	0.2530		

	SS	*df*	MS	F	F_0.05(2,2)_	Significant
Variance analysis						
A	0.0111	2	0.0056	56.0000	19.0000	*
B	0.0534	2	0.0267	267.0000		**
C	0.1361	2	0.0681	681.0000		****
D	0.0989	2	0.0495	495.0000		***
Error	0.0001	2				

From Table 2, the order of the trace elements affecting Pp-7250 multiplication is: Zn^2+^ > Mn^2+^ > Fe^2+^ > Cu^2+^. The experimental results show that the two factors of Zn^2+^ and Mn^2+^ have a significant effect on the multiplication. The optimum fermentation process of Pp-7250 is A1B3C1D2. That is, the mass concentration of Cu^2+^ is 30 mg/L, the mass concentration of Fe^2+^ is 100 mg/L, the mass concentration of Zn^2+^ is 50 mg/L, and the mass concentration of Mn^2+^ is 50 mg/L. It can be seen from the analysis of variance in Table 2 that Zn^2+^ has the most significant effect on the multiplication of Pp-7250, followed by Mn^2+^, Fe^2+^ and Cu^2+^. This result is consistent with the result of the extreme analysis of the orthogonal experiment results. Therefore, the optimal combination for promoting Pp-7250 multiplication is: 30 mg/L Cu^2+^, 100 mg/L Fe^2+^, 50 mg/L Zn^2+^ and 50 mg/L Mn^2+^.

### Optimization of C source and N source conditions for large-scale culture of *Paenibacillus polymyxa* Pp-7250

**Fig. 2:**
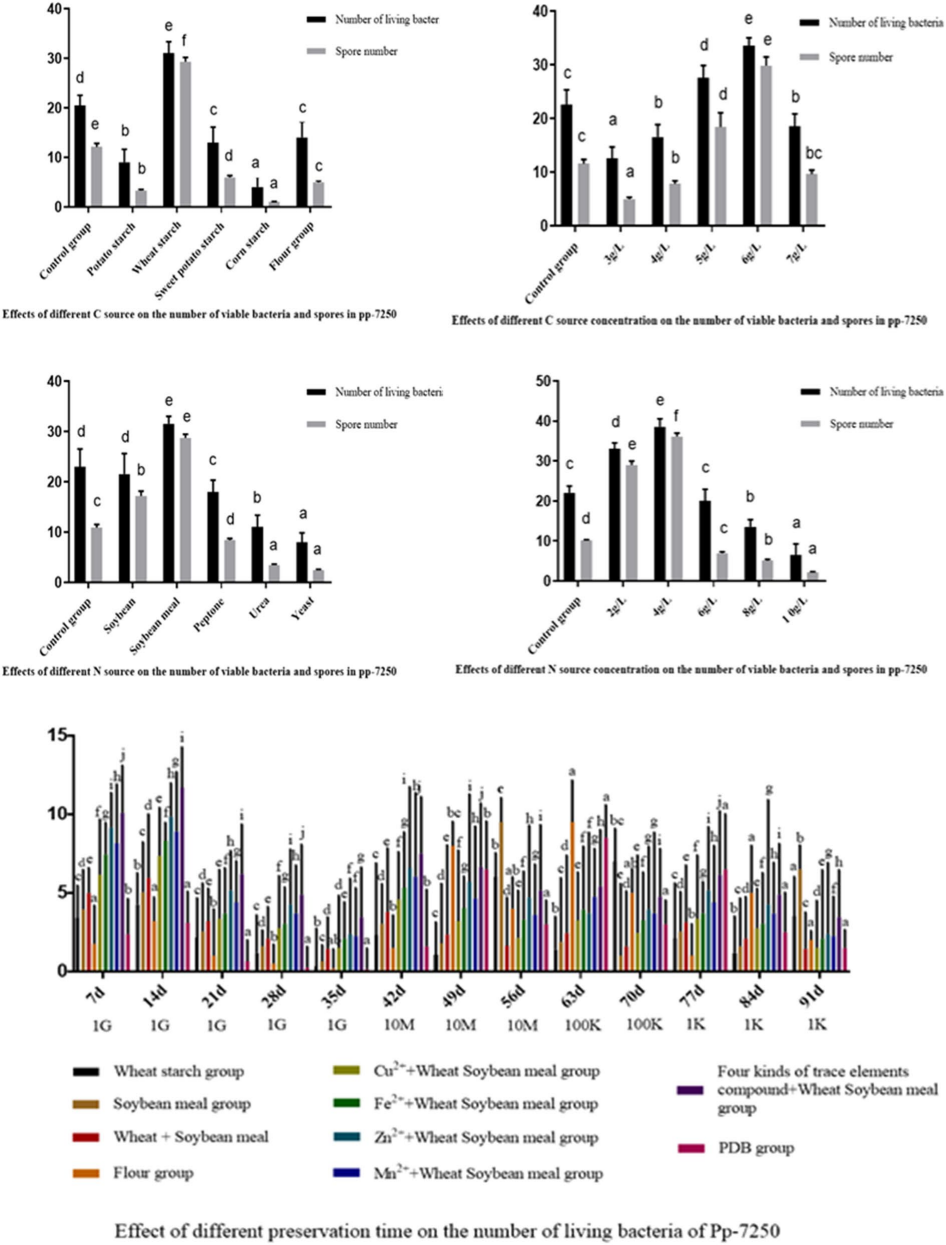
The best C and N source types, concentration screening and the effect of different preservation time on the number of living bacteria of Pp-7250. a, *Mucor* sp.; b, *Fusarium* sp.; c, *Geotrichum candidum*; d, *Pseudomonas*

The single factor studies was conducted on the types and concentrations of different carbon sources, types and concentrations of nitrogen sources. The optimum medium composition for the large-scale culture of Pp-7250 strain was: glucose 20 g/L, wheat starch 6 g/L, and soybean meal 4 g/L (Fig. 2). In this experiment, the conditions for preservation were selected at normal temperature and pressure, which was consistent with the condition in practical application. The effect of the mass concentration of four trace elements on the number of living bacteria in the storage of Pp-7250 at room temperature indicated that Cu^2+^, Fe^2+^, Mn^2+^ and Zn^2+^ could significantly increase the number of living bacteria of Pp-7250 (*P *<0.05). Among them, Zn^2+^ had the best promoting effect, which increased by more than 68% compared with the PDB control group. The combination of four trace elements was better than single trace elements in promoting the number of living bacteria of Pp-7250 (*P *<0.05). The number of living bacteria in the four trace elements compound + wheat soybean meal group remained above 10^7^ CFU/mL in the 8 week. Because some bacteria adapted to the preservation environment in the process of storage, the mortality of the bacteria tended to be stable after 11 weeks, and some of the bacteria could still survive (Fig. 2).

### Effect of four trace elements on Pp-7250 biocontrol

**Fig. 3:**
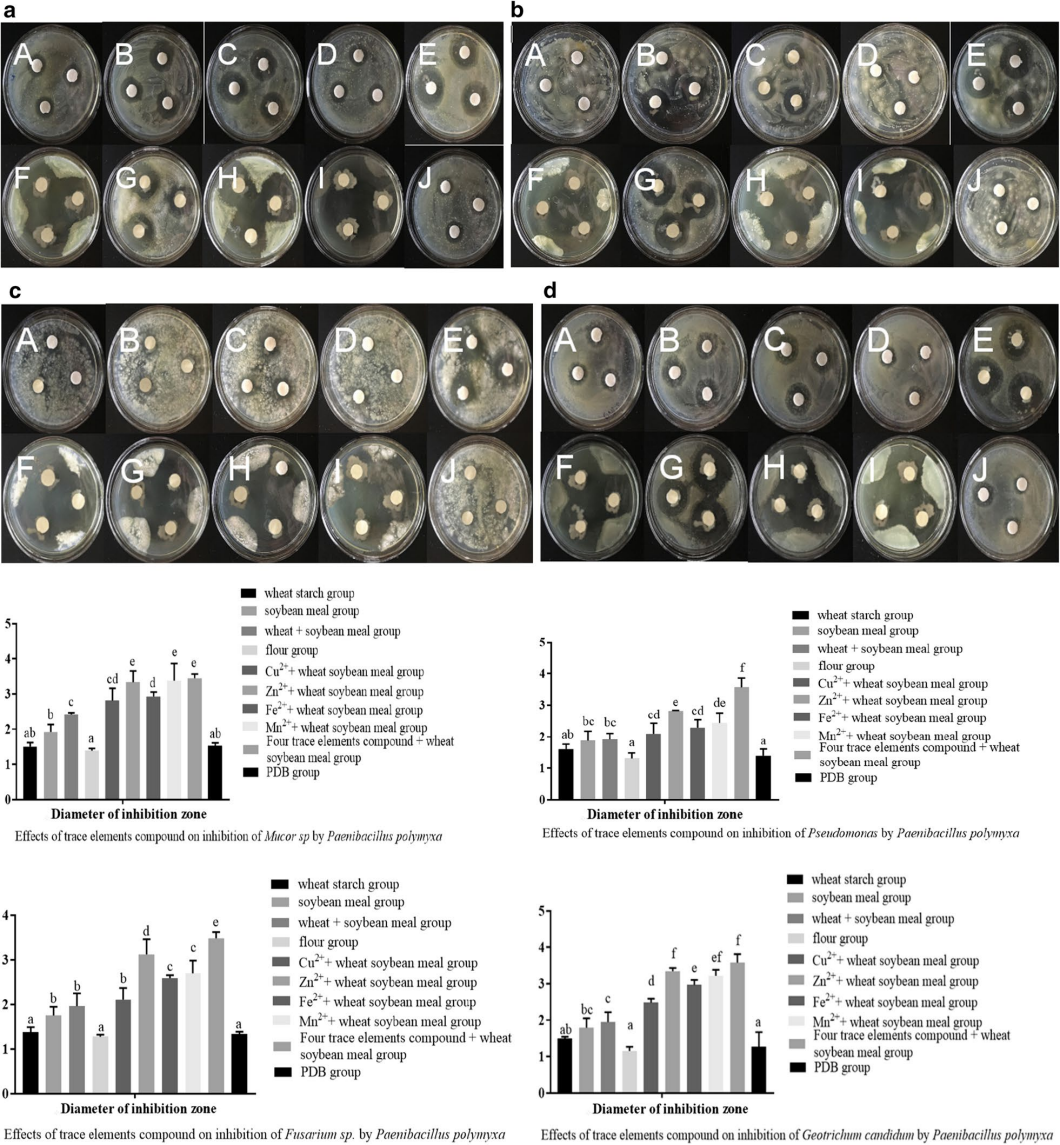
Effects of four trace elements on the antibacterial activity of Pp-7250. a, *Muco*r sp.; b, *Fusarium* sp.; c, *Geotrichum candidum*; d, *Pseudomonas*

**Fig. 4:**
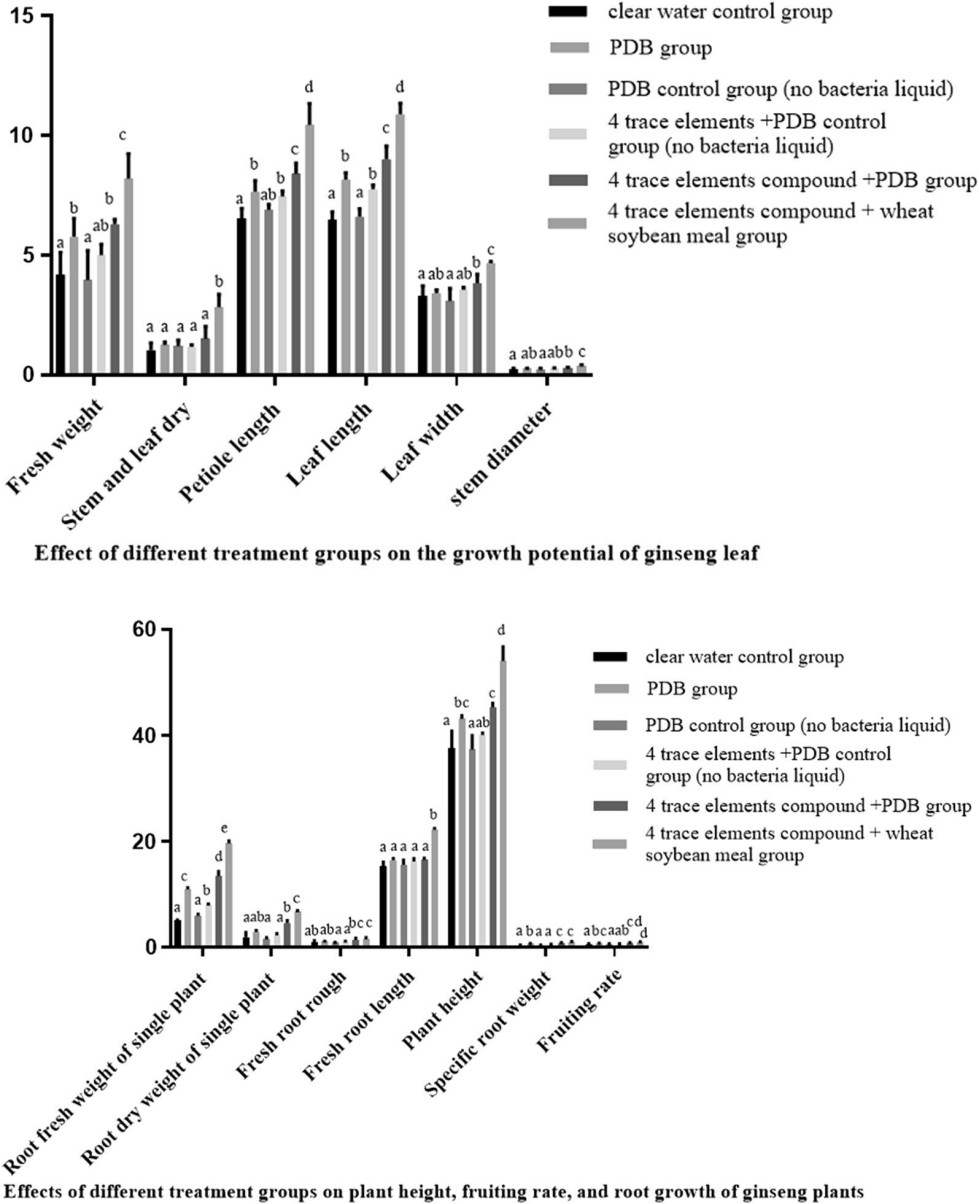
Effects of different treatment groups on leaf growth potential, plant height, fruiting rate and root growth of ginseng

The effects of four trace elements compound on the inhibition of *Mucor*, *Fusarium*, *Geotrichum*, *Pseudomonas* by *Paenibacillus polymyxa* have been shown in the figure, and the results are consistent. Compared with other single trace elements, Zn^2+^ had the best antimicrobial activity against four kinds of pathogenic bacteria at the optimum concentration. The antimicrobial activity of four trace elements compound was better than single trace elements. In general, the effects of trace elements compound are more significant (Fig. 3). At present, scholars at home and abroad generally believe that the plant height, stem diameter and leaf width of ginseng are directly related to the yield and quality of ginseng. The plant height represents the quality of the plant, the stem diameter is related to the stability of the plant quality, and the leaf width directly reflects the effect of photosynthesis on the plant. This study showed that the combination of four trace elements plus wheat soybean meal had the best effect on promoting growth of ginseng leaves, and the fresh weight of single stem and leaf increased by 29.61% compared with the PDB control group. The dry weight of single stem and leaf increased by 55.40%. The petiole length increased by 26.79%; leaf length increased by 24.98%; leaf width increased by 26.87%; stem diameter increased by 31.40% (*P *<0.05). The specific root weight is the ratio of root weight to root length. A high specific root weight indicates that the crop has strong resistance. On the contrary, the lower the root weight, the more sensitive the plant is. Therefore, specific root weight is an important index to measure the quality of crop growth. This study showed that the combination of four trace elements plus wheat soybean meal had the best effect on root growth of ginseng, and the fresh weight of the single root increased by 57.15% compared with the PDB group. The dry weight of single root of ginseng increased by 29.70%. The fruit rate of ginseng increased by 21.62% and the height of ginseng plant increased by 44.32%. The crude root of ginseng increased by 25.69%, the fresh root length of ginseng increased by 20.12%, and the specific root weight of ginseng increased by 26.52% (*P *<0.05) (Fig. 4).

### Effect of different treatment groups on the content of ginseng roots and leaf saponins transformed by *Paenibacillus polymyxa*

**Fig. 5:**
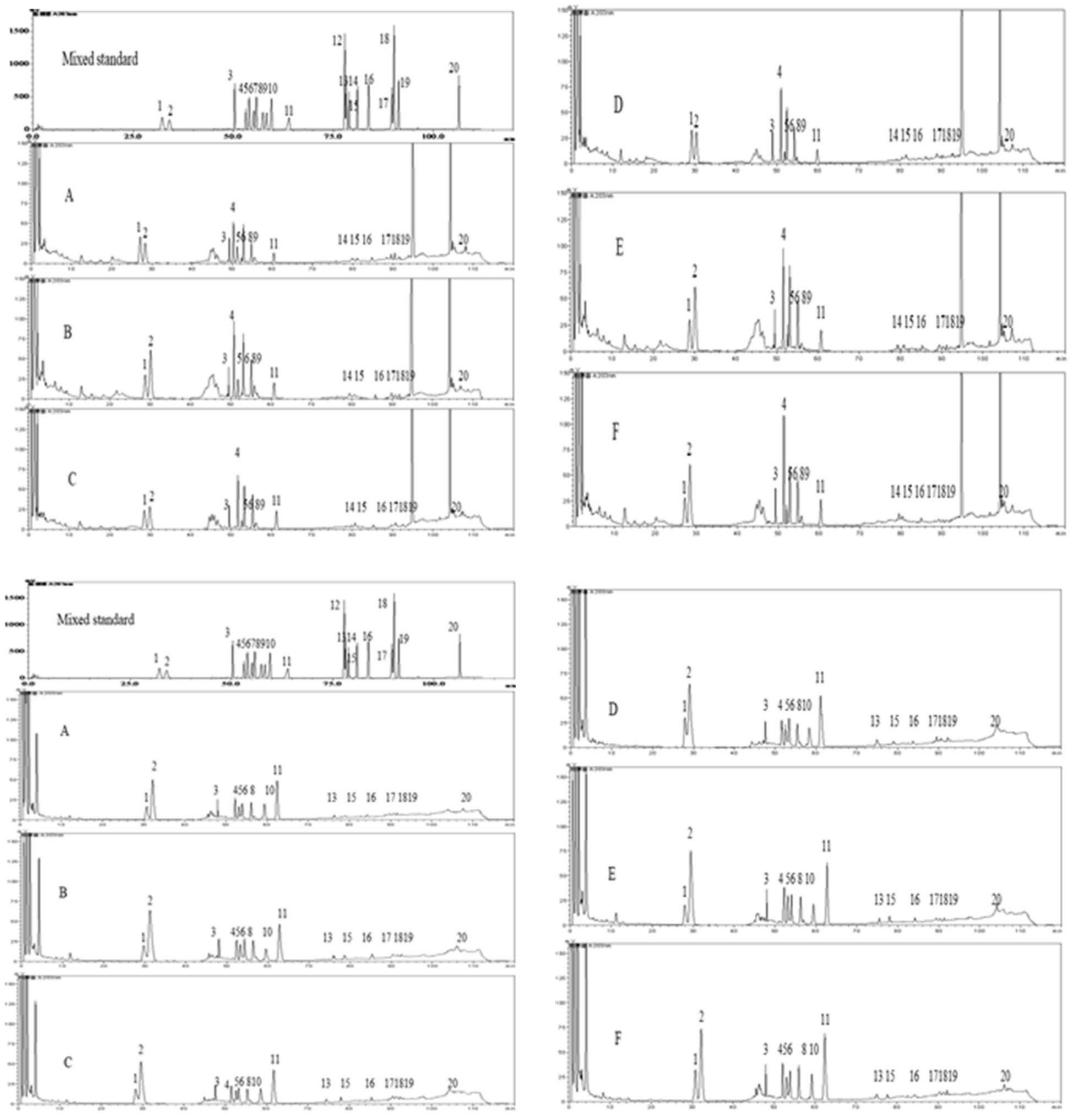
Mixed standard and ginseng root and leaf samples HPLC chromatogram. 1–20 are ginsenoside Rg1, Re, Rf, Rb1, Rg2, Rc, Rh1, Rb2, Rb3, F1, Rd, Rk3, F2, Rh4, Rg3, Protopanaxatriol, Compound K, Rg5, Rh2, Protopanaxadiol. A, Clear water control group; B, PDB group; C, PDB control group (without bacteria solution); D, Four kinds of trace elements + PDB control group (without bacterial liquid); E, Four kinds of trace element compound + PDB Group; F, Four kinds of trace elements compound + wheat soybean meal group

As shown in the figure and table, the four trace elements in the transformation system could significantly increase the content of ginseng roots and leaf saponin transformed by *Paenibacillus polymyxa.* The effects of four kinds of trace elements compound with wheat soybean meal group were significantly better than those of PDB group and four kinds of trace elements +PDB control group (without bacteria solution) (*P *<0.05). Compared with the PDB group, the content of ginseng root saponins Rg1, Re, Rg2, Rc, Rb2, Rb3, Rd and 20 kinds of ginsenoside addition and value increased significantly in the four kinds of trace elements compound + wheat soybean meal group (*P* < 0.05); the content of ginseng leaf saponins Rg1, Rb1, F1, Protopanaxatriol, CK and 20 kinds of ginsenoside addition and value also increased significantly in the four kinds of trace elements compound + wheat soybean meal group (*P *< *0.05*) (Fig. 5) (Table 3).

**Table 3:** Changes of the content of ginseng root and leaf saponins transformed by *Paenibacillus polymyxa* under different treatment groups

	A	B	C	D	E	F
Content of ginseng root saponins transformed by *Paenibacillus polymyxa* under different treatment groups						
Rg1	0.2738 ± 0.0667^a^	0.3433 ± 0.0263^ab^	0.2951 ± 0.0719^ab^	0.2758 ± 0.0467^a^	0.4068 ± 0.0977^bc^	0.5078 ± 0.1303^c^
Re	0.2016 ± 0.0831^a^	0.3552 ± 0.0507^ab^	0.2973 ± 0.0686^a^	0.2836 ± 0.0759^a^	0.4868 ± 0.1206^bc^	0.5710 ± 0.2180^c^
Rf	0.0621 ± 0.0040^a^	0.0856 ± 0.0205^ab^	0.0704 ± 0.0163^a^	0.0654 ± 0.0130^a^	0.0991 ± 0.0152^ab^	0.1189 ± 0.0597^b^
Rb1	0.2052 ± 0.0387^a^	0.3301 ± 0.0715^abc^	0.2559 ± 0.0491^a^	0.2753 ± 0.0317^ab^	0.4040 ± 0.0461^bc^	0.4218 ± 0.1877^c^
Rg2	0.0213 ± 0.0149^a^	0.0353 ± 0.0093^ab^	0.0207 ± 0.0177^a^	0.0258 ± 0.0050^a^	0.0517 ± 0.0178^bc^	0.0679 ± 0.0114^c^
Rc	0.0646 ± 0.0124^a^	0.0828 ± 0.0092^a^	0.0760 ± 0.0321^a^	0.0737 ± 0.0136^a^	0.1162 ± 0.0278^b^	0.1469 ± 0.0265^b^
Rh1	ND	ND	ND	ND	ND	ND
Rb2	0.1013 ± 0.0542^a^	0.1684 ± 0.0434^a^	0.1463 ± 0.0679^a^	0.1549 ± 0.0312^a^	0.2563 ± 0.0231^b^	0.3452 ± 0.0493^c^
Rb3	0.0154 ± 0.0066^a^	0.0254 ± 0.0075^a^	0.0183 ± 0.0031^a^	0.0198 ± 0.0021^a^	0.0383 ± 0.0036^b^	0.0440 ± 0.0156^b^
F1	ND	ND	ND	ND	ND	ND
Rd	0.1255 ± 0.0183^ab^	0.1385 ± 0.0108^ab^	0.1219 ± 0.0195^a^	0.1229 ± 0.0109^a^	0.1492 ± 0.0187^bc^	0.1653 ± 0.0113^c^
Rk3	ND	ND	ND	ND	ND	ND
F2	ND	ND	ND	ND	ND	ND
Rh4	0.0012 ± 0.0002^a^	0.0020 ± 0.0012^a^	0.0016 ± 0.0003^a^	0.0030 ± 0.0022^a^	0.0020 ± 0.0012^a^	0.0026 ± 0.0028^a^
Rg3	0.0035 ± 0.0010^a^	0.0089 ± 0.0037^a^	0.0074 ± 0.0025^a^	0.0078 ± 0.0036^a^	0.0065 ± 0.0030^a^	0.0073 ± 0.0048^a^
Protopan axatriol	0.0054 ± 0.0027a	0.0059 ± 0.0040^a^	0.0048 ± 0.0033^a^	0.0046 ± 0.0035^a^	0.0048 ± 0.0021^a^	0.0055 ± 0.0040^a^
CK	0.0039 ± 0.0024^a^	0.0044 ± 0.034^ab^	0.0046 ± 0.0023^ab^	0.0054 ± 0.0031^ab^	0.0061 ± 0.0020^ab^	0.0105 ± 0.0074^b^
Rg5	0.0087 ± 0.0032^a^	0.0182 ± 0.0190^ab^	0.0210 ± 0.0206^ab^	0.0222 ± 0.0167^ab^	0.0276 ± 0.0184^ab^	0.0413 ± 0.0139^b^
Rh2	0.0045 ± 0.0026^a^	0.0055 ± 0.0038^a^	0.0057 ± 0.0043^a^	0.0055 ± 0.0040^a^	0.0096 ± 0.0084^ab^	0.0143 ± 0.0011^b^
Protopan axadiol	0.0037 ± 0.0046^a^	0.0070 ± 0.0048^ab^	0.0057 ± 0.0055^ab^	0.0050 ± 0.0046^a^	0.0056 ± 0.0049^ab^	0.0140 ± 0.0078^b^
Total saponins	1.1014 ± 0.1430^a^	1.6163 ± 0.1034^b^	1.3523 ± 0.1414^a^	1.3506 ± 0.0981^a^	2.0704 ± 0.1986^c^	2.4842 ± 0.2698^d^
Content of ginseng leaf saponins transformed by *Paenibacillus polymyxa* under different treatment groups						
Rg1	0.1973 ± 0.0523^a^	0.2807 ± 0.0310^ab^	0.2299 ± 0.0437^ab^	0.2782 ± 0.0615^ab^	0.3280 ± 0.0989^bc^	0.3963 ± 0.0403^c^
Re	0.2346 ± 0.1960^a^	0.5740 ± 0.2654^abc^	0.4246 ± 0.2002^ab^	0.4916 ± 0.3641^ab^	0.7432 ± 0.1534^bc^	0.8904 ± 0.1488^c^
Rf	0.0664 ± 0.0117^a^	0.1052 ± 0.0222^b^	0.0566 ± 0.0320^a^	0.0613 ± 0.0093^a^	0.1187 ± 0.0123^b^	0.1234 ± 0.0240^b^
Rb1	0.1287 ± 0.0547^a^	0.1307 ± 0.0297^a^	0.1332 ± 0.0128^a^	0.1328 ± 0.0182^a^	0.1667 ± 0.0152^ab^	0.1846 ± 0.0291^b^
Rg2	0.0613 ± 0.0393^a^	0.0881 ± 0.0295^ab^	0.0710 ± 0.0289^a^	0.0679 ± 0.0145^a^	0.0966 ± 0.0649^b^	0.1048 ± 0.0174^b^
Rc	0.0581 ± 0.0247^a^	0.0686 ± 0.0351^ab^	0.0511 ± 0.0419^a^	0.0454 ± 0.0556^a^	0.0806 ± 0.0343^b^	0.0917 ± 0.0104^b^
Rh1	ND	ND	ND	ND	ND	ND
Rb2	0.0764 ± 0.0447^a^	0.1417 ± 0.0212^bc^	0.0960 ± 0.0222^ab^	0.1034 ± 0.0260^ab^	0.1664 ± 0.0449^c^	0.1779 ± 0.0331^c^
Rb3	ND	ND	ND	ND	ND	ND
F1	0.0178 ± 0.0020^a^	0.0299 ± 0.0077^ab^	0.0227 ± 0.0026^ab^	0.0258 ± 0.0057^ab^	0.0301 ± 0.0042^b^	0.0584 ± 0.0801^c^
Rd	0.1237 ± 0.0382^ab^	0.1440 ± 0.0483^b^	0.1112 ± 0.0494^a^	0.1218 ± 0.0226^a^	0.1408 ± 0.0366^b^	0.1588 ± 0.0292^b^
Rk3	ND	ND	ND	ND	ND	ND
F2	0.0017 ± 0.0010^a^	0.0025 ± 0.0005^a^	0.0026 ± 0.0011^a^	0.0020 ± 0.0008^a^	0.0029 ± 0.0005^a^	0.0031 ± 0.0016^a^
Rh4	ND	ND	ND	ND	ND	ND
Rg3	0.0049 ± 0.0005^a^	0.0073 ± 0.0025^bc^	0.0064 ± 0.0015^ab^	0.0067 ± 0.0006^ab^	0.0080 ± 0.0013^bc^	0.0093 ± 0.0012^c^
Protopan axatriol	0.0014 ± 0.0002^a^	0.0039 ± 0.0011^ab^	0.0025 ± 0.0007^ab^	0.0034 ± 0.0013^ab^	0.0046 ± 0.0019^b^	0.0075 ± 0.0033^c^
CK	0.0019 ± 0.0019^a^	0.0035 ± 0.0046^a^	0.0025 ± 0.0010^a^	0.0027 ± 0.0013^a^	0.0032 ± 0.0024^a^	0.0076 ± 0.0035^b^
Rg5	0.0008 ± 0.0006^a^	0.0009 ± 0.0006^a^	0.0009 ± 0.0005^a^	0.0010 ± 0.0004^a^	0.0011 ± 0.0006^a^	0.0014 ± 0.0007^a^
Rg5	0.0050 ± 0.0035^a^	0.0062 ± 0.0017^a^	0.0053 ± 0.0021^a^	0.0053 ± 0.0014^a^	0.0064 ± 0.0025^a^	0.0083 ± 0.0018^a^
Protopan axadiol	0.0037 ± 0.0006^a^	0.0045 ± 0.0022^a^	0.0036 ± 0.0026^a^	0.0041 ± 0.0012^a^	0.0054 ± 0.0017^a^	0.0088 ± 0.0067^a^
Total saponins	0.9836 ± 0.0606^a^	1.5915 ± 0.0532^b^	1.2200 ± 0.0675^ab^	1.3532 ± 0.0244^ab^	1.9026 ± 0.0885^c^	2.2322 ± 0.0797^d^

A, Clear water control group; B, PDB group; C, PDB control group (without bacteria solution); D, four kinds of trace elements + PDB control group (without bacterial liquid); E, four kinds of trace element compound + PDB Group; F, four kinds of trace elements compound + wheat soybean meal group (lowercase letters indicate that *P *< 0.05 to reach significant levels)

### Effects of four trace elements on Pp-7250 colonization and rhizosphere bacterial community

**Fig. 6:**
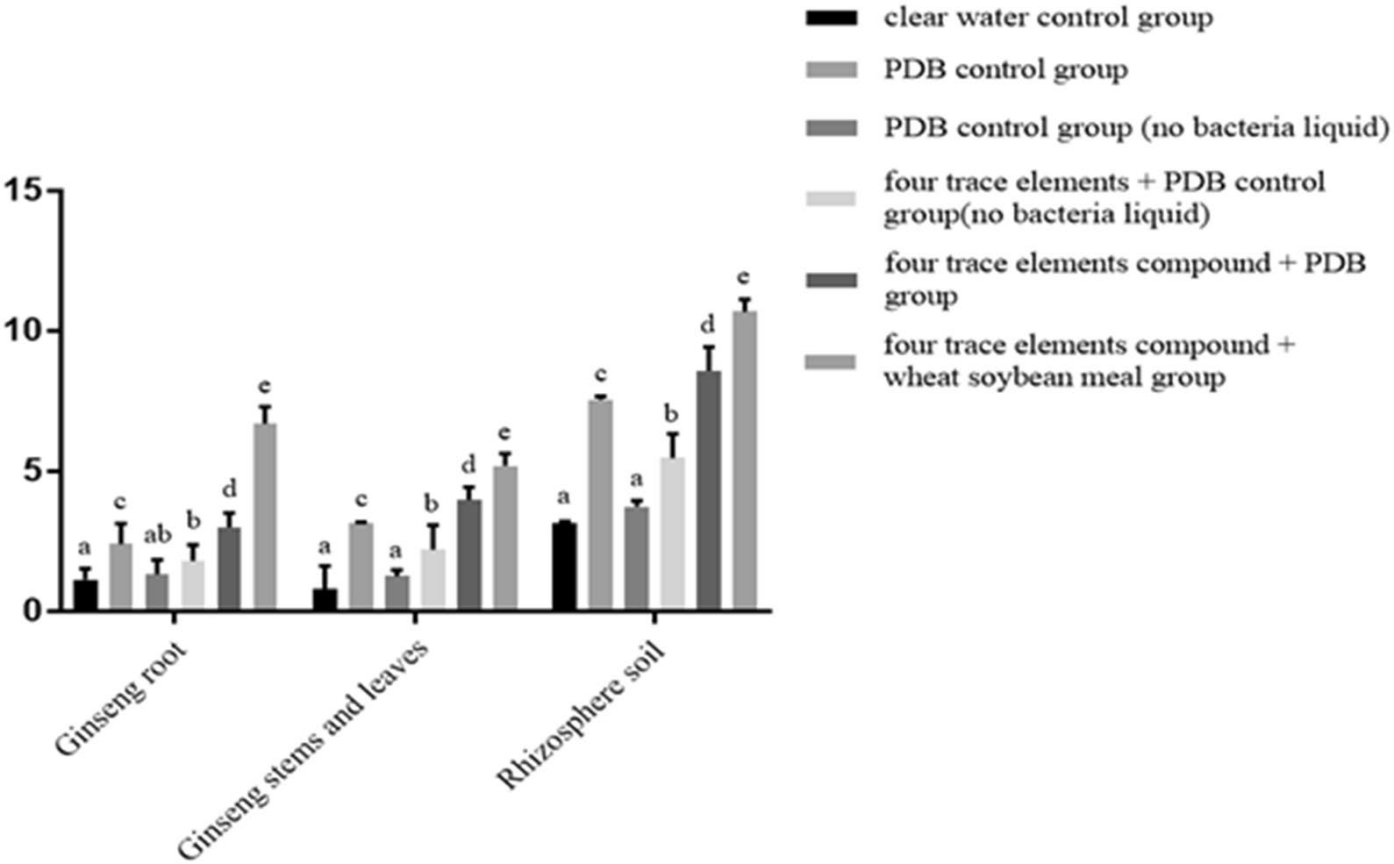
Effect of trace elements on the colonization of Pp-7250 in ginseng roots, leaves and rhizosphere soil

The key to play a stable role in biocontrol bacteria is to successfully colonize the plant surface, body and plant rhizosphere soil, and its colonization ability determines the size of its role. *Paenibacillus polymyxa* is one of the dominant populations of plant rhizosphere soil and has strong anti-stress and disease-resistance ability. Whether it can be successfully colonized on the surface of plants, in vivo and plant rhizosphere soil of plants determines its control effect. This study found that Pp-7250 could effectively colonized the roots, leaves and root surface soil of ginseng. Under the conditions of culture of four trace elements + bacteria solution + wheat soybean meal group, the highest colonization amounts of *Paenibacillus polymyxa* in the roots, leaves, and root surface soil were 6.69 × 10^6^ CFU/g, 5.20 × 10^6^ CFU/g, and 10.7 × 10^6^ CFU/g, respectively; Compared with the PDB group, the amount of colonization increased by 63.98%, 39.81%, 29.44% (Fig. 6).

**Fig. 7:**
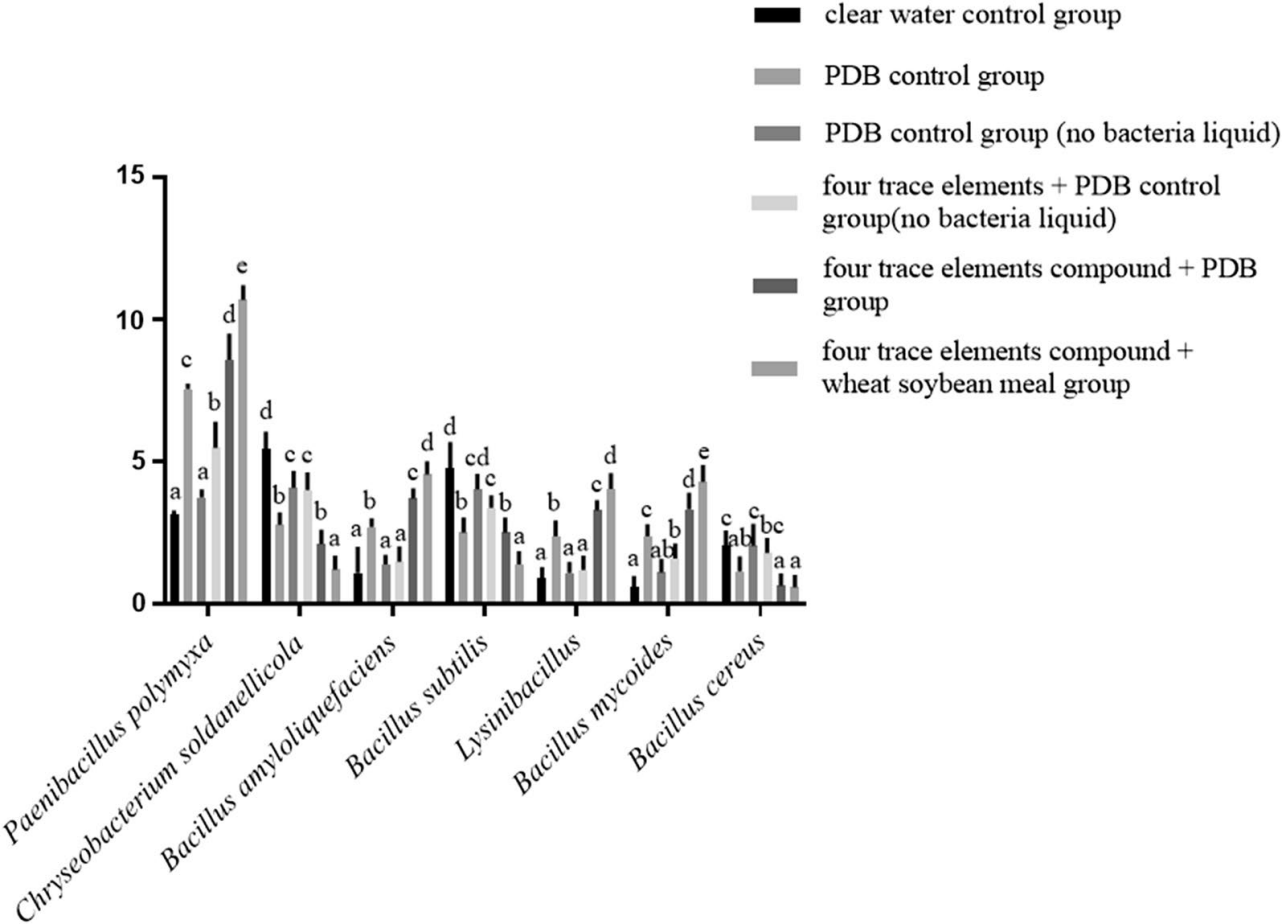
Effect of trace elements on the rhizosphere bacterial community of ginseng

The composition of soil bacteria microorganisms is an important part of the rhizosphere soil microecosystem, and its composition and activity are important for the maintenance and transformation of soil fertility. In this study, strains with different colony morphology were isolated from ginseng rhizosphere soil, according to the observation of colony morphology and PCR amplification technology, the sequences obtained were compared by BLAST. The biological classification status of each strain was determined. They were *Bacillus amyloliquefaciens*, *Bacillus mycoides*, *Chryseobacterium soldanellicola*, *Bacillus subtilis*, *Lysinibacillus*, *Bacillus cereus* and *Paenibacillus polymyxa.* The experimental study showed that the four kinds of trace elements compound plus wheat soybean meal group bacteria liquid promoted the number of probiotics *Paenibacillus polymyxa*, *Bacillus amyloliquefaciens*, *Lysinibacillus* and *Bacillus mycoides.* The inhibition of *Bacillus subtilis*, *Bacillus cereus*, and *Bacillus subtilis* in the rhizosphere soil of ginseng was significant (*P *<0.05) (Fig. 7).

## Discussion

Trace elements are an indispensable nutrient factor for normal metabolism of microorganisms. They are part of the active centers of various enzymes and participate in the synthesis and metabolism of substances in microorganisms. It has an important influence on the stability of biological biomacromolecules and cell structure in microorganisms; it controls the REDOX potential of cells; it can also serve as an energy substance for the proliferation of certain microorganisms. A large number of researchers have found that by adding trace elements, plant diseases can be reduced; the survival conditions of microorganisms can be improved, and their own proliferation can be promoted or their secretion of production-promoting biomass can be increased to promote plant growth; It can affect the ability of microorganisms to convert ginsenosides (Cui et al. 2016; Han et al. 2010; Liu et al. 2010). Findings in this study that the appropriate concentrations of trace elements and their combination are beneficial to the proliferation of *Paenibacillus polymyxa*, and have a certain reference value for the application in the prevention and control of plant diseases. It is suggested that the suitable range of trace elements should be considered in the practical application of *Paenibacillus polymyxa*. With the improvement of modern microbiological industrial production technology system, the development of related microbial action and related products has become a research hotspot. It is closely related to human food safety, living environment and species diversity. *Bacillus* probiotics have become the main content of development in recent years due to its unique form of spores, which has better commercial stability than other probiotics. Due to the urgent need for a large amount of fermentation broth, sufficient samples are prepared to meet the needs of microbial dosage form research and field efficacy trials. Therefore, it is imperative to develop a large-scale culture of *Bacillus* by optimizing the C source and N source culture medium. But in practical applications, *Paenibacillus polymyxa* may not be used immediately after large-scale culture, it will take some time if it is transported to a distant place. With the increase of time, the number, activity, and effect will may be reduced, affecting the subsequent applications. Therefore, the effect of different preservation time on the viable count of *Paenibacillus polymyxa* is determined. Finding that the number of living bacteria of *Paenibacillus polymyxa* cultured in the combination of four trace elements plus wheat pods decreased less with the increase of time. *Paenibacillus polymyxa* is harmless to humans and animals, can produce a variety of antibiotics and enzymes, has a broad-spectrum antibacterial activity and strong anti-stress ability (Tupinamba et al. 2008). Many good traits of *Paenibacillus polymyxa* biocontrol strains have been applied to field control research, not only can promote the growth of crops such as soybeans, rice, cucumber and others, increase their yield, but also show a good biocontrol effect. Therefore, how to ensure the growth state of the bacteria by adding trace elements in the Pre-fermentation and Mid-fermentation to improve the ability of preventing diseases and promoting plant growth of *Paenibacillus polymyxa* Pp-7250 is particularly important. So in this study, the effect of *Paenibacillus polymyxa* on ginseng growth in different treatment groups is investigated by field experiments. Our laboratory has already confirmed the ability of *Paenibacillus polymyxa* to transform ginsenosides. On this basis, four trace elements were added in this experiment. The results are encouraging. All four trace elements promote the transformation of ginsenosides by *Paenibacillus polymyxa*, it is speculated that the activity of glycosyltransferase may be increased due to the appropriate mass concentration of Cu^2+^; Fe^2+^ catalyzes the activity of key enzymes in the process of the glycolysis; Mn^2+^ activates the activity of farnesyl pyrophosphate synthase; Zn^2+^ is related to the expression of key enzyme genes in the synthesis of ginsenosides (Khorolragcha et al. 2014; Wang et al. 2018; Lu et al. 2017; Xing et al. 2012; Jung et al. 2014; Kim et al. 2015). However, the mechanism of its action needs to be further studied. The key to the stability of biocontrol bacteria lies in the successful colonization of plant surface, body and plant rhizosphere soil (Patel et al. 2018; Syed et al. 2018; Thokchom et al. 2017). Due to the influence of external environmental conditions, biocontrol bacteria in competition with the soil indigenous microorganisms is not easy to hold a dominant position in the long-term survival and colonization, which greatly affects their actual disease-preventing effect. Thus, it is of great significance to further explore the colonization characteristics of biocontrol bacteria in the application process and the effect of biocontrol strains on the rhizosphere microbial community of host plants. This experiment mainly focused on the colonization of ginseng roots, stems and leaves, and rhizosphere soils of *Paenibacillus polymyxa* cultured in different treatment groups, and its effect on the ginseng rhizosphere bacterial community. In order to provide theoretical basis and technical guidance for biological control and application development of *Paenibacillus polymyxa* in field. The results of this experiment show that the combination of four trace elements plus wheat soybean meal group bacteria liquid can promote the number of probiotics *Paenibacillus polymyxa*, *Bacillus amyloliquefaciens*, *Bacillus mycoides* and *Lysinibacillus.* It is speculated that there may be symbiotic phenomena among several microorganisms, which can promote each other, grow together, increase crop yield, and effectively resist and control plant diseases and insect pests. However, the quantity of *Bacillus subtilis*, *Bacillus cereus*, *Chryseobacterium soldanellicola* is significantly inhibited in the root soil of ginseng. Speculated that there may be competition, and the demand for nutrition and surrounding environment among microorganism is relatively close. The competition for the living space and nutrients around the roots of plants inhibits the proliferation of microorganisms (Cosme and Wurst 2013; Zhang et al. 2014; Zamioudis and Pieterse 2012; Lou et al. 2018).

## Abbreviations

C: carbon
N: nitrogen
Pp-7250: *Paenibacillus polymyxa* 7250
PCR: polymerase chain reaction
PDA: potato dextrose agar
rDNA: ribosome deoxyribonucleic acid
DNA: deoxyribonucleic acid
BLAST: basic local alignment search tool
NCBI: National Center for Biotechnology Information

## References

[CR1] Akladious SA, Mohamed HI (2018). Ameliorative effects of calcium nitrate and humic acid on the growth, yield component and biochemical attribute of pepper (*Capsicum annuum*) plants grown under salt stress. Sci Hortic-Amsterdam.

[CR2] Bai L, Cui JQ, Jie W, Cai B (2015). Analysis of the community compositions of rhizosphere fungiin soybeans continuous cropping fields. Microbiol Res.

[CR3] Blamey J, Chiong M, López C, Smith E (1999). Optimization of the growth conditions of the extremely thermophilic microorganisms *Thermococcus celer*, and *Pyrococcus woesei*. J Microbiol Meth.

[CR4] Cheng AC, Lin HL, Shiu YL, Tyan YC, Liu CH (2017). Isolation and characterization of antimicrobial peptides derived from *Bacillus subtilis*, E20-fermented soybean meal and its use for preventing *Vibrio*, infection in shrimp aquaculture. Fish Shellfish Immun.

[CR5] Chinheya CC, Yobo KS, Laing MD (2017). Biological control of the rootknot nematode, *Meloidogyne javanica* (Chitwood) using *Bacillus*, isolates, on soybean. Biol Control.

[CR6] Cosme M, Wurst S (2013). Interactions between arbuscular mycorrhizal fungi, rhizobacteria, soil phosphorus and plant cytokinin deficiency change the root morphology, yield and quality of tobacco. Soil Biol Biochem.

[CR7] Cui L, Wu SQ, Zhao CA, Yin CR (2016). Microbial conversion of major ginsenosides in ginseng total saponins by *Platycodon grandiflorum* endophytes. J Gins Res.

[CR8] Cui JQ, Bai L, Liu XR, Jie W, Cai B (2018). Arbuscular mycorrhizal fungal communities in the rhizosphere of a continuous cropping soybean system at the seedling stage. Braz J Microbiol.

[CR9] Ding H, Zhang F, Cao Y, Long LV, Muying D (2012). Improvement of potato dextrose agar medium preparation. Chin Brewing.

[CR10] Finch EA, Caruso T, Engl C (2018). Effects of *Paenibacillus polymyxa*, inoculation on below-ground nematode communities and plant growth. Soil Biol Biochem.

[CR11] Gao YG, Liu JT, Ji Q, Zhao Y, Zang P, He ZM, Zhu HY, Zhang LX (2018). Anti-tumor activity and related mechanism study of *Bacillus Polymyxa* Transformed *Panax ginseng* C. A. Mey. Process Biochem..

[CR12] Ge XY, He CE, Li T, Zhu OY (2015). Effect of *Bacillus subtilis* and *Pseudomonas fluorescens* on growth of greenhouse tomato and rhizosphere microbial community. J Northeast Agric Univ.

[CR13] Ghasemi S, Ahmadzadeh M (2013). Optimisation of a cost-effective culture medium for the large-scale production of *Bacillus subtilis* UTB96. Arch Phytopathol Plant Protect.

[CR14] Gowtham HG, Murali M, Singh SB, Lakshmeesha TR, Murthy KN, Amruthesh KN, Niranjana SR (2018). Plant growth promoting rhizobacteria *Bacillus amyloliquefaciens*, improves plant growth and induces resistance in chilli against anthracnose disease. Biol Control.

[CR15] Gupta KG, Sidhu R, Yadav NK (2010). Effect of monovalent and divalent ions upon the germination of *Bacillus* spores in the presence of nisin. J Food Sci.

[CR16] Han Y, Sun B, Jiang B, Hu X, Spranger MI (2010). Microbial transformation of ginsenosides Rb1, Rb3 and Rc by *Fusarium sacchari*. J Appl Microbiol.

[CR17] He Y, Chen Z, Liu X, Wang C, Lu W (2014). Influence of trace elements mixture on bacterial diversity and fermentation characteristics of liquid diet fermented with probiotics under air-tight condition. PLoS ONE.

[CR18] Jeong JH, Kim JN, Wee YJ, Ryu HW (2010). The statistically optimized production of poly (γ-glutamic acid) by batch fermentation of a newly isolated *Bacillus subtilis* RKY3. Bioresour Technol.

[CR19] Ji Q, Gao YG, Zhao Y, He ZM, Zhang LX (2015). Determination of ginsenosides by *Bacillus polymyxa* conversion and evaluation on pharmacological activities of the conversion products. Process Biochem.

[CR20] Jia RB, Guo WL, Zhou WB, Jiang YJ, Zhu FF (2017). Screening and identification of *Monascus* strain with high TMP production and statistical optimization of its culture medium composition and liquid state fermentation conditions using response surface methodology (RSM). Biotechnol Biotec Eq..

[CR21] Jung SC, Kim W, Park SC, Jeong J, Park MK (2014). Two ginseng UDP-glycosyl transferases synthesize ginsenoside Rg3 and Rd. Plant Cell Physiol.

[CR22] Khorolragcha A, Kim Y-J, Rahimi S, Sukweenadhi J, Jang MG, Yang DC (2014). Grouping and characterization of putative glycosyltransferase genes from *Panax ginseng* Meyer. Gene.

[CR23] Kim YJ, Zhang D, Yang DC (2015). Biosynthesis and biotechnological production of ginsenosides. Biotechnol Adv.

[CR24] Lazarovits G, Turnbull A, Johnston-Monje D (2014). Plant health management: biological control of plant pathogens. J Food Sci..

[CR25] Li L, Ma Y (2014). Effects of metal ions on growth, β-oxidation system, and thioesterase activity of *Lactococcus lactis*. J Dairy Sci.

[CR26] Liu L, Gu LJ, Zhang DL, Wang Z, Wang CY (2010). Microbial conversion of rare ginsenoside Rf to 20(*S*)-protopanaxatriol by *Aspergillus niger*. Biosci Biotechnol Biochem.

[CR27] Lou Y, Guo Q, Peng C, Shi MD, Li HY, Li X, Xue QH, Lai HX (2018). Effects of three *Bacillus* strains on growth promoting and rhizosphere soil microflora of tomato. Chin J Appl Ecol.

[CR28] Lu C, Zhao SJ, Wang XS (2017). Functional regulation of a UDP-glucosyltransferase gene (*Pq3*-*O*-*UGT1*) by RNA interference and overexpression in *Panax quinquefolius*. Plant Cell Tiss Org.

[CR29] Mahmood M (2010). Trace elements for growth and bulbiformin production by *Bacillus subtilis*. J Appl Microbiol.

[CR30] Memoli V, Eymar E, García-Delgado C, Esposito F, Panico SC, Marco AD, Barile R, Maisto G (2018). Soil element fractions affect phytotoxicity, microbial biomass and activity in volcanic areas. Sci Total Environ.

[CR31] Mercado-Flores Y, Cárdenas-Álvarez IO, Rojas-Olvera AV, Pérez-Camarillo JP, Leyva-Mir SG, Anducho -Reyes MA (2014). Application of *Bacillus subtilis* in the biological control of the phytopathogenic fungus *Sporisorium reilianum*. Biol Control.

[CR32] Midhuna SJ, Neethu S, Vysakh A, Arun D, Radhakrishnan EK, Jyothis M (2017). Antibacterial activity and probiotic characterization of autochthonous *Paenibacillus polymyxa* isolated from *Anabas testudineus* (Bloch, 1792). Microb Pathogenes.

[CR33] Mosquera S, González JLM, Orduz S, Escobar VV (2014). Multiple response optimization of *Bacillus subtilis* EA-CB0015 culture and identification of antifungal metabolites. Biocatal Agric Biotechnol.

[CR34] Nagano N, Taoka Y, Honda D, Hayashi M (2013). Effect of trace elements on growth of marine eukaryotes, tharaustochytrids. J Biosci Bioeng.

[CR35] Odeniyi OA, Adeola OJ (2017). Production and characterization of polyhydroxyalkanoic acid from *Bacillus thuringiensis* using different carbon substrates. Int J Biol Macromol.

[CR36] Park YH, Mishra RC, Yoon S, Kim H, Park C, Seo ST, Bae H (2018). Endophytic *Trichoderma citrinoviride* isolated from mountain-cultivated ginseng (*Panax ginseng*) has great potential as a biocontrol agent against ginseng pathogens. J Gins Res..

[CR37] Patel JK, Madaan S, Archana G (2018). Antibiotic producing endophytic *Streptomyces* spp. colonize above-ground plant parts and promote shoot growth in multiple healthy and pathogen-challenged cereal crops. Microbiol Res..

[CR38] Piromyou P, Noisangiam R, Uchiyama H, Tittabutr P, Boonkerd N, Teaumroong N (2013). Indigenous microbial community structure in rhizosphere of Chinese Kale as affected by plant growth-promoting Rhizobacteria inoculation. Pedosphere.

[CR39] Prabakaran G, Balaraman K (2006). Development of a cost-effective medium for the large scale production of *Bacillus thuringiensis var israelensis*. Biol Control.

[CR40] Prabakaran G, Hoti SL (2008). Influence of amino nitrogen in the culture medium enhances the production of δ-endotoxin and biomass of *Bacillus thuringiensis var israelensis* for the large-scale production of the mosquito control agent. J Ind Microbiol Biotechnol.

[CR41] Prakasham RS, Rao CS, Rao RS, Lakshmi GS, Sarma PN (2007). l-Asparaginase production by isolated *Staphylococcus* sp.—6A: design of experiment considering interaction effect for process parameter optimization. J Appl Microbiol.

[CR42] Raza W, Wu H, Shen Q (2010). Use of response surface methodology to evaluate the effect of metal ions (Ca^2+^, Ni^2+^, Mn^2+^, Cu^2+^) on production of antifungal compounds by *Paenibacillus polymyxa*. Bioresour Technol.

[CR43] Ropek D, Para A (2002). The effect of heavy metal ions and their complexions upon the growth, sporulation and pathogenicity of the entomopathogenic fungus *Verticillium lecanii*. J Invertebr Pathol.

[CR44] Ruming Z, Yi L, Wenhua L, Ping S, Songsheng Q (2003). Effect of Sm^3+^ ion on growth of *Bacillus thuringiensis* by microcalorimetry. Biol Trace Elem Res.

[CR45] Schnider U, Keel C, Blumer C, Troxler J, Défago G (1995). Amplification of the housekeeping sigma factor in *Pseudomonas fluorescens* CHA0 enhances antibiotic production and improves biocontrol abilities. J Bacteriol.

[CR46] Sharifazizi M, Harighi B, Sadegh A (2017). Evaluation of biological control of *Erwinia amylovora*, causal agent of fire blight disease of pear by antagonistic bacteria. Biol Control.

[CR47] Sharma R, Sindhu S, Sindhu SS (2018). Suppression of *Alternaria* blight disease and plant growth promotion of mustard (*Brassica juncea* L.) by antagonistic rhizospherebacteria. Appl Soil Ecol.

[CR48] Siahmoshteh F, Esfahani ZH, Spadaro D, Ghahfarokhi MS, Abyaneh MR (2018). Unraveling the mode of antifungal action of *Bacillus subtilis* and *Bacillus amyloliquefaciens* as potential biocontrol agents against aflatoxigenic *Aspergillus parasiticus*. Food Control.

[CR49] Syed SAR, Singh E, Pieterse CMJ, Schenk PM (2018). Emerging microbial biocontrol strategies for plant pathogens. Plant Sci.

[CR50] Tabbene O, Slimene IB, Djebali K, Mangoni ML, Urdaci MC (2009). Optimization of medium composition for the production of antimicrobial activity by *Bacillus subtilis* B38. Biotechnol Progr.

[CR51] Thokchom E, Thakuria D, Kalita MC, Sharma CK, Talukdar NC (2017). Root colonization by host-specific rhizobacteria alters indigenous root endophyte and rhizosphere soil bacterial communities and promotes the growth of mandarin orange. Eur J Soil Biol.

[CR52] Touré Y, Ongena M, Jacques P, Guiro A, Thonart P (2004). Role of lipopeptides produced by *Bacillus subtilis* GA1 in the reduction of grey mould disease caused by *Botrytis cinerea* on apple. J Appl Microbiol..

[CR53] Trchounian K, Poladyan A, Trchounian A (2016). Optimizing strategy for *Escherichia coli* growth and hydrogen production during glycerol fermentation in batch culture: effects of some heavy metal ions and their mixtures. Appl Energ.

[CR54] Tupinamba G, Da SAJ, Souto-Padron T, Seldin L, Alviano DS (2008). Antimicrobial activity of *Paenibacillus polymyxa* SCE2 against some mycotoxin-producing fungi. J Appl Microbiol.

[CR55] Wang Q, Chen S, Zhang J, Sun M, Liu Z, Yu Z (2008). Co-producing lipopeptides and poly-gamma-glutamic acid by solid-state fermentation of *Bacillus subtilis* using soybean and sweet potato residues and its biocontrol and fertilizer synergistic effects. Bioresour Technol.

[CR56] Wang DD, Jin Y, Wang C, Kim YJ, Perez ZEJ, Baek NI, Mathiyalagan R, Markus J, Yang DC (2018). Rare ginsenoside Ia synthesized from F1 by cloning and overexpression of the UDP-glycosyltransferase gene from *Bacillus subtilis*: synthesis, characterization, and in vitro melanogenesis inhibition activity in BL6B16 cells. J Gins Res.

[CR57] Xing Z, Long Y, He S, Liang N, Li B (2012). Molecular cloning of farnesyl diphosphate synthase from *Eleutherococcus senticosus* and its bioinformatics and expression analysis. Chin J Chin Mater Med.

[CR58] Yang Y, Meng F, Gao Y, Zhang L (2016). Simultaneous determination of twenty ginsenosides in ginseng preparations by HPLC. Food Sci.

[CR59] Yang A, Zeng S, Yu L, He M, Yang YY, Zhao XZ, Jiang CL, Hu D, Song B (2018). Characterization and antifungal activity against pestalotiopsis, of a fusaricidin-type compound produced by *Paenibacillus polymyxa*, Y-1. Pestic Biochem Phys.

[CR60] Zamioudis C, Pieterse CM (2012). Modulation of host immunity by beneficial microbes. Mol Plant Microbe In.

[CR61] Zhang XH, Wang K, Zhu TH, Cui ZF (2013). Optimization of medium and fermentation conditions for the production of antifungal substance by *Bacillus amyloliquefaciens* BW-13. J Zhejiang Univ Tech.

[CR62] Zhang F, Duan TY, Yan FY, Fang L (2014). Advances in the interactions of arbuscular mycorrhizal fungi and rhizosphere microorganisms. Pratac Sci.

